# The tumour-associated carbonic anhydrases CA II, CA IX and CA XII in a group of medulloblastomas and supratentorial primitive neuroectodermal tumours: an association of CA IX with poor prognosis

**DOI:** 10.1186/1471-2407-10-148

**Published:** 2010-04-18

**Authors:** Kristiina Nordfors, Joonas Haapasalo, Miikka Korja, Anssi Niemelä, Jukka Laine, Anna-Kaisa Parkkila, Silvia Pastorekova, Jaromir Pastorek, Abdul Waheed, William S Sly, Seppo Parkkila, Hannu Haapasalo

**Affiliations:** 1 Department of Pathology, Tampere University Hospital, Tampere, Finland; 2 Department of Neurosurgery, Helsinki University Central Hospital, Helsinki, Finland; 3 Department of Medical Biochemistry and Genetics, University of Turku, Turku, Finland; 4 Department of Pathology, Turku University Hospital, Turku, Finland; 5 Department of Neurology, Tampere University Hospital, Tampere, Finland; 6 Centre of Molecular Medicine, Institute of Virology, Slovak Academy of Sciences, Bratislava, Slovak Republic; 7 Edward A. Doisy Department of Biochemistry and Molecular Biology, Saint Louis University School of Medicine, St Louis, MO, USA; 8 Institute of Medical Technology and School of Medicine, University of Tampere and Tampere University Hospital, Tampere, Finland

## Abstract

**Background:**

Medulloblastomas (MBs) and supratentorial primitive neuroectodermal tumours (PNETs) are the most common highly aggressive paediatric brain tumours. In spite of extensive research on these tumours, there are only few known biomarkers or therapeutic target proteins, and the prognosis of patients with these tumours remains poor. Our aim was to investigate whether carbonic anhydrases (CAs), enzymes commonly overexpressed in various tumours including glioblastomas and oligodendrogliomas, are present in MBs and PNETs, and whether their expression can be correlated with patient prognosis.

**Methods:**

We determined the expression of the tumour-associated carbonic anhydrases CA II, CA IX and CA XII in a series of MB/PNET specimens (*n *= 39) using immunohistochemistry.

**Results:**

Endothelial CA II, cytoplasmic CA II, CA IX and CA XII were expressed in 49%, 73%, 23% and 11% of the tumours, respectively. CA II was detected in the neovessel endothelium and the tumour cell cytoplasm. CA IX was mainly expressed in the tumour cells located in perinecrotic areas. CA XII showed the most homogenous distribution within the tumours. Importantly, CA IX expression predicted poor prognosis in both univariate (*p *= 0.041) and multivariate analyses (*p *= 0.016).

**Conclusions:**

We suggest that CA IX should be considered a potential prognostic and therapeutic target in MBs and PNETs.

## Background

Medulloblastomas (MBs) and primitive neuroectodermal tumours (PNETs) are classified as embryonal tumours of the central nervous system (CNS) and histologically correspond to WHO grade IV [1]. One viewpoint postulates that these tumours show a common ontogeny, arising from related progenitor cells that have the potential for divergent neuroepithelial differentation. However, in recent years molecular genetic analyses have demonstrated different genetic profiles for these tumours [1]. It has been proposed that MBs originate from the neoplastic transformation of granule cell precursors in the cerebellum via deregulation of molecular pathways involved in normal cerebellar development [2,3]. Correspondingly, PNETs arise in the cerebral hemispheres, brain stem or spinal cord. The neuroepithelial tumour cells of a PNET may be undifferentiated or poorly differentiated. In addition, the tumour cells may have aberrant differentiations, including neuronal, astrocytic and ependymal lines.

MB is the most common childhood malignant tumour of the central nervous system and accounts for 12-25% of all paediatric CNS tumours. It is very rare in adults, accounting for only 0.5-1% of brain tumours [4]. The main defective cell signalling pathways involved in the development of MB include the Hedgehog and Wnt pathways, but the exact molecular mechanisms contributing to tumourigenesis in both MB and PNET are still poorly understood [5].

MBs are sensitive to chemotherapy and radiation, but surgical resection continues to be the most effective treatment [6,7]. Patients with PNET undergo a similar treatment process to patients with MB [8]. There has been a marked improvement in the 5-year survival rate in MB patients, as the survival percentage has improved from 2-30% in the 1970s to 60-70% currently [1,4]. Unfortunately, the current clinical staging does not effectively identify the patients whose tumours will be resistant to chemotherapy and radiation. To individualise therapies and minimise side-effects of aggressive treatments, we need to overcome the major challenge of identifying the high- and low-risk patients. While the prognosis for patients with MB has improved, children with PNET have an even worse prognosis than patients with MB. Currently, the 5-year survival rate for patients with PNET is 24-38% [9,10].

The carbonic anhydrases (CAs) are zinc-containing metalloenzymes that catalyse the reversible hydration of carbon dioxide (CO_2 _+ H_2_O ⇔ HCO_3_^- ^+ H^+^), and, thus, participate in the maintenance of pH homeostasis in the body [11-14]. The mammalian α-CA gene family encodes at least thirteen enzymatically active isoforms with different structural and catalytic properties, and twelve of these are expressed in human tissues [15]. CA isozymes II, IX and XII have been associated with neoplastic processes, and they are potential histological and prognostic biomarkers of certain tumours, including diffuse astrocytomas [12,16,17]. CA II is the most widely distributed member of the CA gene family, being present in virtually every human tissue and organ. It is catalytically one of the most efficient enzymes known [18]. It is present to some extent in malignant cells, and, interestingly, it has been recently shown to be ectopically expressed in the endothelial cells of tumour neovessels [17,19]. Transmembrane enzyme, CA IX, was first recognised as a novel tumour-associated antigen expressed in several types of human carcinomas as well as in normal gastrointestinal tissue [12,20,21]. It has been functionally linked to cell adhesion, differentiation, proliferation and oncogenic processes [12,22], and its enzymatic activity is comparable to CA II [23]. Another transmembrane CA isozyme, CA XII, was first found in normal kidney tissue and renal cell carcinoma [24,25]. Later studies have shown that it is expressed in several other tumours, but also in some normal organs such as the colon and uterus [26,27].

CA IX and XII seem to be regulated by similar mechanisms, as transcription of these isozymes is induced in tumours under hypoxic conditions through hypoxia inducible factor-1 alpha (HIF-1α)-mediated pathways [28]. Even though very little is known about the regulation of CA II expression, it is unlikely that HIF-1α is involved. High expression of CA II, IX and XII in certain tumours, such as astrocytomas and oligodendrogliomas [16,17,29,30], has suggested that these enzymes may functionally participate in the invasion process, which is facilitated by acidification of the extracellular space [31]. In favour of this hypothesis, it has been shown in vitro that CA inhibitors can reduce the invasion capacity and proliferation of cancer cells [32-34].

To our knowledge, this is the first study to assess the expression of tumour-associated CAs in MBs and PNETs. Here we evaluate the expression of CA II, IX and XII in association with the patient age, survival and molecular pathologic features such as apoptosis and expression of c-erbB2, MIB-1 and bcl-2.

## Methods

### Study material

Brain tumour samples were obtained from 35 patients (15 females and 20 males) with either MBs or supratentorial PNETs who were operated on at the University Hospitals of Tampere and Turku, Finland, from1989-2005. The term supratentorial PNET is used as a synonym for CNS PNET, not otherwise specified [1]. MBs were observed in 28 patients and supratentorial PNETs in 7 patients. In addition, there were four patients with a recurrent tumour (two MBs: recurrence after 8 and 29 months in the cerebellum; two supratentorial PNETs: recurrences after 9 and 71 months in the brain stem and left frontal lobe, respectively). Taken together, our material included 39 surgical tumour samples. The age of the patients varied from newborn to 68 years (median = 7.4, mean ± SD = 14.4 ± 17.2), Table 1.

**Table 1:** Patient characteristics in different tumour subtypes and the correlation between them.

	MB	PNET	All primary tumours	p-value
Age (mean, years)	15.0 ± 17.3	11.9 ± 17.8	14.4 ± 17.2	0.343*

Sex				
Females	10	5	15	
Male	18	2	20	0.088**

Therapy				
Surgery only	3	2	3	
Surgery +radiation	3	1	4	
Surgery+chemotherapy	3	0	5	
Surgery+radiation+chemo	19	4	23	0.540**

* Mann-Whitney test

** chi-square test

In the early nineties eight-drugs-in-one -protocol and later vincristine, lomustine and prednisolon were widely used also for MB and PNETs [35]. Later the treatment in older children (over three years of age) started with radiation therapy with weekly vincristin doses (craniospinal dose 36 Gy and total tumor dose 54-55 Gy), and after irradiation a chemotherapy protocol using cisplatin, CCNU and vincristine was applied [36]. The later protocol is still in use. Children under three years of age have been treated with multidrug chemotherapy protocols from Childrens Cancer Group (USA) or German HIT-SKK-group generally without radiation therapy. Of the 35 patients, 4 received preoperative chemotherapy and/or radiation therapy, and these four patients all had a recurrent tumour. The tumours were radically resected if possible, and most patients were also treated with postoperative chemo- and/or radiotherapy as follows: three patients received surgery only, five patients were post-operatively treated with chemotherapy, four underwent radiotherapy and twenty-three patients received both postoperative chemotherapy and radiotherapy, Table 1.

The overall survival was known for 35 patients, and 17 patients were alive and 18 patients dead at the end of the follow-up period. The 5-year survival for our patients was 46% in the total tumour material, 39% in MBs and 71% in PNETs.

All the material was gathered from surgical operations. For immunohistochemistry, the brain tumour specimens were fixed immediately in 4% phosphate-buffered formaldehyde and processed into paraffin blocks. Haematoxylin and eosin-stained slides of the tumours were evaluated by two experienced neuropathologists, and the histopathological typing and grading were carried out according to WHO criteria [1]. Following the typing and grading of the specimens, a neuropathologist (HH) pinpointed one histologically representative area from each tumour with a high cellular proliferation index (as assessed by Ki-67 (MIB-1) staining) [37], and this area was then inserted into a multitissue block. The blocks were constructed with a custom-built instrument (Beecher Instruments, Silver Spring, MD) and the diameter of the tissue cores was 2 mm.

### Immunohistochemistry

The monoclonal antibody M75, recognising the N-terminal domain of human CA IX, has been described previously [20,21]. The rabbit anti-human CA XII antiserum against the secretory form of CA XII has been characterised by Karhumaa et al. [27]. Rabbit antiserum against human CA II has also been produced and characterised previously [38]. Normal rabbit serum (NRS) was used for control staining.

Immunohistochemical staining for CA II, CA IX and CA XII were performed using an automated immunostaining system with the Power Vision+ Poly-HRP IHC Kit reagents (ImmunoVision Technologies, Burlingame, CA). Briefly, the sections were: (a) rinsed in a wash buffer; (b) treated with 3% H_2_O_2 _in ddH_2_O for 5 min and rinsed in a wash buffer; (c) blocked with the Universal IHC Blocking/Diluent for 30 min and rinsed in a wash buffer; (d) incubated for 30 min with the rabbit anti-human CA II serum, rabbit-anti human CA XII serum, monoclonal M75 antibody or NRS diluted 1:2000 (rabbit sera) or 1:1000 (M75) in Universal IHC Blocking/Diluent; (e) rinsed in a wash buffer for 5 min three times; (f) incubated in Poly-HRP-conjugated anti-rabbit/mouse IgG for 30 min and rinsed in a wash buffer for 5 min three times; (g) incubated in a DAB (3,3' diaminobenzidine tetrahydrochloride) solution (one drop DAB solution A and one drop DAB solution B with 1 ml ddH_2_O) for 6 min; (h) rinsed with ddH_2_O; (i) treated with CuSO_4 _for 5 min to enhance the signal and (j) rinsed with ddH_2_O. All procedures were carried out at room temperature. The sections were finally examined and photographed with a Zeiss Axioskop 40 microscope (Carl Zeiss; Göttingen, Germany).

The staining reactivities for CA II, CA IX and CA XII were scored from the multitissue- blocks on a scale from 0 to 3 as follows: 0, no reaction; 1, weak reaction (< 10% positive cells); 2, moderate reaction (10-30% positive cells); 3, strong reaction (>30% positive cells). Due to the sample size, the staining results were categorised into two groups: negative staining was considered as CA-negative and weak, moderate and strong staining were considered as CA-positive.

The section preparation, immunostaining and analysis of apoptosis (TUNEL-labelling) [39] and the expression of c-erbB-2, p53 [40] and bcl-2 [41] were done as previously described.

### Statistical analysis

All statistical analyses were performed using SPSS 15.0 for Windows (Chicago, IL). The significance of the associations was defined using the chi-square test, the Mann-Whitney test and the Kruskal-Wallis test. A log rank test, Kaplan-Meier curves and Cox multivariate regression analysis were used in the survival analysis.

### Ethics

The study design was approved by the Ethics committee of Tampere University Hospital and the National Authority for Medicolegal Affairs.

## Results

**Figure 1:**
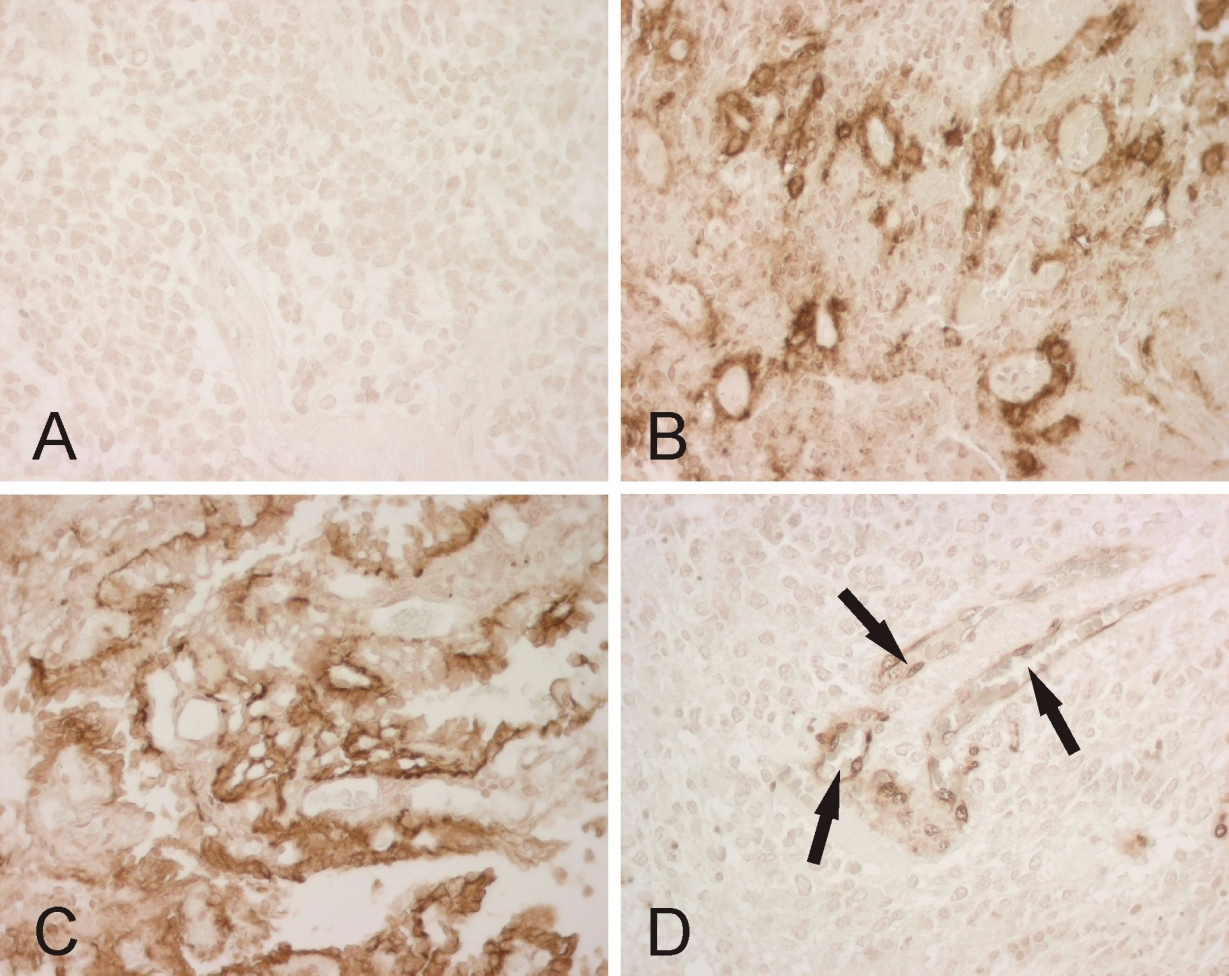
**Representative immunostaining of CA enzymes in MBs**. Panel A shows no immunoreaction for CA IX, whereas the tumour in panel B is strongly positive. Panel C demonstrates CA XII-positive immunoreactivity in tumour cells. In panel D, CA II-positive immunostaining is confined to the endothelium of small blood vessels (arrows). All magnifications ×400.

Immunohistochemical staining of CA II, CA IX and CA XII in tumour specimens is shown in Figure 1. CA II showed two distinct staining patterns: the endothelium of neovessels and the cytoplasm of MB/PNET cells. Of all tumours, 49% (*n *= 18, 12 MBs/6 PNETs) stained positively for CA II in the tumour endothelium (32% strong, 11% moderate and 6% weak staining). Positive cytoplasmic CA II staining in tumour cells was found in 73% (*n *= 27, 20 MBs/7 PNETs) of the cases (11% strong, 38% moderate and 24% weak staining).

CA IX and CA XII were less frequently expressed than CA II in the tumour samples: 23% (*n *= 9, 8 MBs/1 PNET) of the tumours were positive for CA IX (3% strong, 13% moderate and 7% weak staining), and only 11% (*n *= 4, 3 MBs/1 PNET) of the tumours were positive for CA XII (3% strong, 3% moderate and 5% weak staining). The CA IX-specific antibody stained perinecrotic areas in most of the tumours in which necrosis was visible. The CA XII was more homogenously distributed than CA IX, consistent with the results obtained previously in other tumours such as ovarian tumours [42].

Since MBs/PNETs are rare tumours and the availability of the specimens was limited to 39, the positively stained tumours (scores 1-3) were pooled for most statistical analyses, including the studies on patient survival. There was no significant correlation in the co-expression of CA II, IX and XII in the subgroups of MBs and PNETs nor did we found a correlation in the group of all tumours (chi-square test). We also compared CA II, IX and XII expression with various clinical features and molecular markers (Table 2). The expression of the CAs did not correlate with proliferation (MIB-1), apoptosis (chi-square and Mann-Whitney test) or expression of bcl-2, p53 or c-erbB-2 in any of the groups except for the correlation between c-erbB-2 and CA IX in PNETs (*p *= 0.047, chi-square test). Interestingly, CA XII-positive staining correlated with younger patient age (total material *p *< 0.001, MBs *p *< 0.001, chi-square test, Table 2). Added to this, CA IX positivity associated with female gender (total material *p *= 0.048, MBs *p *= 0.023, Table 2, chi-square test). There was no significant difference in the expression of CAs between the primary and recurrent tumours in any of the groups (chi-square test). Moreover, there were no correlation between the tumour type (MBs/PNETs) and CA intensity (chi-square test).

**Table 2:** Association of endothelial and cytoplasmic CA II, CA IX and CA XII immunostaining with clinicopathologic variables in medulloblastomas (MB) and primitive neuroectodermal tumours (PNET).

	endothelial CA II positivity N			cytoplasmic CA II positivity N			CA IX positivity N			CA XII positivity N		
**Primary tumors**	**All**	**MB**	**PNET**	**All**	**MB**	**PNET**	**All**	**MB**	**PNET**	**All**	**MB**	**PNET**

	**33**	**27**	**6**	**33**	**27**	**6**	**35**	**28**	**7**	**34**	**27**	**7**

Age												
< 3 years	5	2	3	5	3	2	1	1	0	4*	3+	1
> 3 years	12	10	2	18	15	3	7	6	1	0	0	0

Gender												
-female	6	3	3	10	7	3	1**	0++	1	1	0	1
-male	11	9	2	13	11	2	7	7	0	3	3	0

Localization												
-cerebellum	12			18			7			3		
-cerebrum	5			5			1			1		

Total material												

MIB-1	**36**	**28**	**8**	**36**	**28**	**8**	**37**	**29**	**8**	**36**	**28**	**8**
-below median	9	6	3	13	10	3	5	4	1	1	1	0
-above median	9	6	3	13	9	4	4	4	0	2	1	1

Apoptosis	**37**	**29**	**8**	**37**	**29**	**8**	**39**	**30**	**9**	**38**	**29**	**9**
-below median	8	5	3	15	11	4	5	5	0	1	1	0
-above median	10	7	3	12	9	3	4	3	1	3	2	1

Bcl-2	**37**	**29**	**8**	**37**	**29**	**8**	**39**	**30**	**9**	**38**	**29**	**9**
-negative	6	4	2	13	9	4	3	2	1	1	0	1
-positive	12	8	4	14	11	3	6	6	0	3	3	0

P53	**37**	**29**	**8**	**37**	**29**	**8**	**39**	**30**	**9**	**38**	**29**	**9**
-negative	15	6	1	23	9	1	7	4	0	3	0	0
-positive	3	6	5	4	11	6	2	4	1	1	3	1

ErbB2	**37**	**29**	**8**	**37**	**29**	**8**	**39**	**30**	**9**	**38**	**29**	**9**
-negative	8	10	5	11	10	5	6	7	0Ψ	0	2	1
-positive	10	2	1	16	2	2	3	1	1	4	1	0

The total number of tumours analysed in each category is in bold.

* p < 0.001, chi-square test

** p = 0.048, chi-square test

+ p < 0.001, chi-square test

++ p = 0.023, chi-square test

Ψ p = 0.047, chi-square test

**Figure 2:**
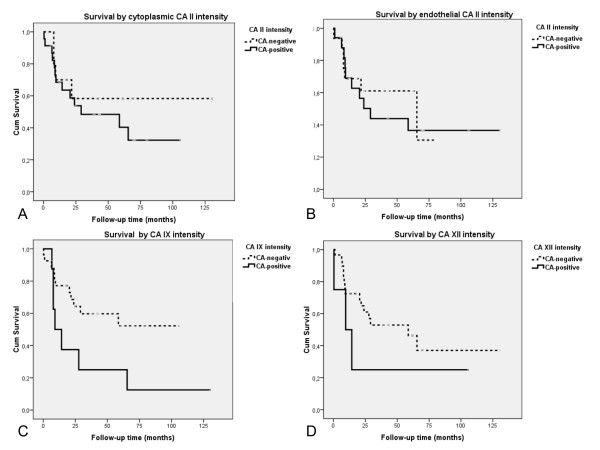
**Kaplan-Meier curves showing overall survival of patients with MB or PNET categorised by: A. tumour cell-associated CA II, B. endothelial CA II, C. CA IX (*p *= 0.041; log-rank test), and D. CA XII immunostaining results**.

All 35 patients with primary MB/PNET were included in the survival analysis (Figure 2). The patients with a CA IX-positive MB/PNET had a worse prognosis than those who had a CA IX-negative tumour (all tumours *p *= 0.041, MBs *p *= 0.030, PNETs *p *= n.s.; log-rank test; Figure 2). We also found a correlation between survival and CA XII staining in patients with MB. The patients with CA XII-positive tumours showed significantly worse prognosis (*p *= 0.010, log-rank test). There was no significant difference in survival time between the histological subgroups (p = 0.463, log-rank test). Of the prognostic indicators used for MBs in the current WHO classification, (2007) the following variables were included into the Cox multivariate survival analysis: patient age, MIB-1 proliferation index, apoptosis index and expression of p53, c-erbB-2 and bcl-2. In addition, the histopathological group (MB vs. supratentorial PNET), CA II, CA IX and CA XII were used in the analysis. These variables were grouped as presented in Table 2. In the Cox analysis, only expression of CA IX (odds ratio 4.31; 95% confidence interval (CI) 1.31 - 14.11; *p *= 0.016) and the apoptosis index (odds ratio 3.29; 95% CI 1.05 - 10.31, *p *= 0.041) were independent prognostic factors. The expression of either CA II and CA XII failed to show any significant association with survival.

## Discussion

In the present study, we demonstrate that several MBs and supratentorial PNETs express the CA isozymes CA II, CA IX and CA XII. According to the univariate survival analysis, expression of CA IX was found to be associated with poor prognosis. Most importantly, the Cox multivariate analysis, which included patient age, tumour cell proliferation, apoptosis rate and several other molecular factors, demonstrated that CA IX expression and apoptotic activity were the only independent prognostic factors. The expression of CA XII in the tumour cells was associated with patient age, as previously reported for the expression of CA XII in patients with diffuse astrocytomas [29]. These findings reflect the fact that patient age is a significant factor that contributes to carcinogenesis by several mechanisms and that tumour phenotypes are different depending on the age of the patient.

The expression of CA II, CA IX and CA XII in the normal nervous system has been investigated in several previous studies. The localization of CA II is well documented in the normal human oligodendrocytes [43]. Based on our previous studies, CA IX is not present in the normal human brain [16]. RT-PCR analysis has shown very weak CA XII mRNA expression in the human brain [29], and in mouse, immunohistochemical staining has located CA XII to the choroid plexus [44].

CA IX has several functions in tumour progression. It has been proposed to have a capacity to modulate E-cadherin-mediated cell adhesion, thus leading to a more aggressive phenotype of malignant cells. In intercellular junctions, CA IX may be linked to the E-cadherin/β-catenin complex, because CA IX co-immunoprecipitated with β-catenin in cultured MDCK cells, a kidney cell line [45]. It is also noteworthy that β-catenin is mutated in some sporadic cases of MB [46]. The presence of CA IX in the E-cadherin/β-catenin complex might contribute by an unknown mechanism to increased invasion and spread of tumour cells. Indeed, embryonal tumours differ from other brain tumours by their tendency to metastasise. In addition, our previous findings in diffusely infiltrating astrocytomas are also in line with the suggested role of CAs in the invasion process. Typically, CA IX-positive astrocytic tumours are highly invasive tumours with an extremely poor prognosis [16]. A recent study by Chiche et al. [47] provided clear evidence that both CA IX and CA XII are functionally involved in tumour growth. In vivo experiments showed that *CA9 *gene silencing alone led to a 40% reduction in xenograft tumour volume, and the silencing of both *CA9 *and *CA12 *resulted in an 85% reduction in tumour volume.

In this study, CA IX was found in the perinecrotic areas of the tumours whenever necrosis was present. A similar hypoxia-associated pattern of CA IX expression has been previously detected in astrocytic tumours [16]. Hypoxia triggers architectural and phenotypic rearrangements of tumour tissue, resulting in the development of necrotic areas surrounded by zones of surviving hypoxic cells. Importantly, these cells often become the most aggressive tumour cells [48], in which CA IX expression is induced by HIF1-α-regulated pathway [28]. Because necrosis is an uncommon feature and is not considered to be a significant prognostic factor in MBs, the induction of CA IX in MBs/PNETs may also involve hypoxia-independent mechanisms. Similarly, in previous studies on gliomas, CA IX expression was seen in tumour cells located in close proximity to the blood vessels [49], and it has been shown that acidosis induces CA IX independently of pericellular hypoxia in glioblastoma cell lines [31]. Based on the previous studies, it has become clear that although hypoxia is the key factor for CA IX induction, there may be other important factors involved. Our text already pointed out that tumor cell acidosis seems to contribute to the expression level [31]. There are several studies where CA IX expression has been correlated to pimonidazole accumulation. The results have shown slightly conflicting results, which may reflect to biological variation between different tumor types and dynamics of tumor hypoxia. However, most results give support for the idea that CA IX follows the pattern of pimonidazole binding [50,51].

We have previously studied the expression of tumour-associated CAs in other types of brain tumours. Endothelial CA II was expressed in the neovessels of astrocytic tumours, in which it was associated with poor prognosis [17]. In addition, we have shown that both CA IX and CA XII are independent prognostic factors in glial tumours [16,29,30]. Based on these findings, CAs may play a central role in the pathogenesis of malignant brain tumours and may represent potential biomarkers for histopathological diagnosis of brain tumours. Although in the total tumour material CA II and CA XII did not reach statistical significance in for use as prognostic indicators, CA II had a similar trend to that of CA IX. Furthermore, CA XII showed a significant correlation with survival in MBs. As discussed above, these differences may be partly explained by regulatory mechanisms. It has been also shown that higher CA IX expression is associated with a more favourable overall survival in some tumours, such as in renal cell carcinoma (RCC) and in acute myeloid leukemia (AML). In RCC the CA IX induction is associated with VHL-mutation and not with hypoxia as in brain tumours [52]. In AML the association has been discussed to be involved with immune system and T-cell response [53]. Hypoxia-induced CA XII is less frequently expressed in MBs/PNETs than in gliomas. Interestingly, CA II was, once again, found in the endothelium of neovessels and, thus, may play an important functional role in tumour metabolism. In melanoma patients, endothelial CA II represents a major target antigen in dendritic cell therapy [19]. Further studies are, therefore, clearly warranted to evaluate the role of CA II as a possible therapeutic target not only in melanoma but also in other forms of cancer, including MBs/PNETs.

CA IX-specific inhibitors would represent ideal candidate molecules for cancer therapy, because CA IX is highly expressed in several cancers while it shows a very limited distribution in normal tissues [22]. Design of isozyme-specific inhibitor has proved to be a great challenge, because the CA active site is quite similar in all active alpha CA isoforms. The recently published crystal structure of CA IX was certainly a major breakthrough that will help to design novel inhibitors with higher specificity [54].

According to our results, apoptosis was another independent prognostic factor in MBs/PNETs, although its role seems to be controversial. On one hand, as we show in this study, a higher apoptotic index is associated with better prognosis [55]. On the other hand, previous studies in which the degree of apoptosis was categorised as 'focal', 'diffuse' or 'extensive' demonstrated a correlation only between survival and focal apoptosis [6]. In addition, it is known that bcl-2 is an inhibitor of apoptosis. In our tissue samples, however, immunoreactivity for bcl-2 did not correlate with better prognosis; although similar results have been reported by others [56]. In our study, apoptosis was an independent prognostic indicator of MBs/PNETs. However, the study material was rather limited due to the fact that MBs and PNETs are rare tumours, and studies will be needed to clarify the association between apoptosis and survival. Added to this, the time period in which the patients were treated, was rather long and treatment protocols varied.

In the future, children diagnosed with MB/PNET will be more accurately stratified based on a combination of clinical variables and molecular profiles. Improved risk stratification will enable individualised therapies, which could be a combination of conventional treatment modalities and novel, targeted therapeutic approaches. These changes will hopefully result in improved survival without a detriment in the quality of life. Several molecular alterations have already been identified in MBs, many of which appear to have prognostic significance.

## Conclusions

Based on our results, CA IX seems to be a promising prognostic marker that should be tested in a larger cohort of MB/PNET patients. The expression of CA IX in some MBs/PNETs suggests that it could be considered a potential therapeutic target, similar to other tumours including acute myeloid leukemia [53] and renal cancer [57]. Furthermore, CA IX could be used for in vivo imaging and as a target molecule for CA inhibitors [12,22].

## Competing interests

The authors declare that they have no competing interests.

## Authors' contributions

KN as the corresponding author gathered the clinical data of the patients, made the statistical analyses, and drafted the first version of the manuscript. JH also participated in the collection of the clinical information, helped with the statistical analyses, and contributed to the writing of the first version of the manuscript. HH was the main organizer of the study. MK provided the clinical knowledge needed and chose the patients for the study at the University Hospital of Turku. AN, JL and HH performed essential microscopic analyses. AP and SPar provided further knowledge on CAs and were in charge of the microscopic analyses on CAs in MBs and PNETs. SPas and JP provided the antibodies against human CA IX, and the antibodies against human CA XII were from the laboratory of AW and WSS. All authors read and approved the final manuscript.

## Pre-publication history

The pre-publication history for this paper can be accessed here:

http://www.biomedcentral.com/1471-2407/10/148/prepub
